# Postnatal care in Rwanda: facilitators and barriers to postnatal care attendance and recommendations to improve participation

**DOI:** 10.29392/joghr.3.e2019032

**Published:** 2019-06-01

**Authors:** Pamela Williams, Nathalie Kayiramirwa Murindahabi, Elizabeth Butrick, David Nzeyimana, Felix Sayinzoga, Bernard Ngabo, Angèle Musabyimana, Sabine F Musange

**Affiliations:** 1 Institute for Global Health Sciences, University of California San Francisco, San Francisco, California, USA; 2 University of Rwanda, College of Medicine and Health Sciences, School of Public Health, Kigali, Rwanda; 3 Maternal, Child and Community Health Division, Rwanda Ministry of Health, Rwanda Biomedical Center, Kigali, Rwanda

## Abstract

**Background:**

Sub-Saharan Africa has the highest rates of neonatal mortality in the world with an estimated 1.2 million deaths within the first 28 days of life. Postnatal care (PNC) can contribute to reductions in morbidity and mortality in mothers and newborns through vital support that identifies danger signs and establishes valuable practices and referral processes.

**Methods:**

This qualitative data was collected as a part the East Africa Preterm Birth Initiative (PTBi-EA) to guide development of a group antenatal (ANC) and PNC model in Rwanda. Key-informant in-depth interviews (IDIs) and focus group discussions (FGDs) were conducted in four districts. Sixteen FGDs with 180 participants and 22 IDIs were completed at the time of thematic saturation.

**Results:**

Four themes highlighted facilitators and barriers to PNC attendance and recommendations to improve participation: 1) There is little awareness in the community of what the PNC package is; PNC 4 in particular is not well understood; 2) PNC visits by community health workers (CHWs) are well accepted and valued; 3) Providers perceive PNC 4 as an added burden to an already high workload; 4) Community structures exist to better disseminate key messages about PNC, but have not yet been effectively utilized.

**Conclusions:**

This qualitative work provides evidence that the PNC package was not initially well understood. Regardless, PNC service delivery performed by CHWs in Rwanda is well accepted and appreciated by the population, providing assurance that the full package has potential to be well utilized and valued by the population.

The importance of care for mother and infant during the postnatal period, defined as the first six weeks after childbirth, has gained increasing attention. Half of all postnatal maternal deaths occur the first week following childbirth (*1*). Sub-Saharan Africa has the highest rates of neonatal mortality in the world with an estimated 1.2 million deaths within the first 28 days of life (*1*). Africa’s population accounts for 16% of the global population, yet 38% of neonatal deaths occur in this region. Furthermore, the region has seen slower improvements in neonatal mortality rates than others (*2*, *3*). Postnatal care (PNC) can contribute to reductions in morbidity and mortality in mothers and newborns through vital support that identifies danger signs and establishes valuable practices and referral processes (*4*).

Common causes of maternal mortality (postpartum hemorrhage, hypertensive disorders, and postpartum sepsis) and newborn deaths (infection, low birth weight, and asphyxia), including those common in preterm infants (sepsis, meningitis, pneumonia, and diarrhea) can nearly all be prevented or treated with appropriate PNC (*5*–*7*). Progress has been made in the reduction of morbidity and mortality in mothers and newborns and the United Nations seeks to continue this trend with new 2030 targets (*8*). Adherence to the 2013 WHO PNC guidelines that prescribe four PNC visits has potential to provide the needed foundation for this progress (*9*).

The majority of neonatal deaths that occur within 48 hours of life can be prevented with care provided immediately after delivery (*10*). PNC should include education promoting immediate and exclusive breastfeeding, hand washing, neonatal temperature management, hygienic cord cleaning, examination for danger signs for the mother and baby, and appropriate referral for care (*10*). Early PNC interventions for newborns include pneumonia case management and referral, breastfeeding support, hypothermia prevention and management, and kangaroo care (*1*, *11*). In addition to these services, specialized PNC monitoring is recommended for preterm, low-birth weight, or infants born to HIV-infected mothers and other high risk cases (*7*). Barriers to the provision of PNC previously recorded in the literature include: mother and family members’ health literacy, influence of sociocultural beliefs and practices, distance to health center, and health providers’ workload and its link to care quality (*12*–*14*).

The Republic of Rwanda provides strong support for maternal and child health interventions. The nation has dramatically reduced its maternal and newborn mortality rates, increased the use of modern contraception, and nearly all women deliver at a healthcare facility (*15*–*19*). Despite this headway, maternal and newborn mortality and morbidity continue: newborn deaths during the PNC period make up about one-third of all child mortality (*13*). According to the Rwanda Demographic Health Survey (DHS), 57% of postpartum women and 81% of newborns do not receive PNC services (*19*).

Numerous infrastructure and health workforce developments have improved PNC utilization in Rwanda. Public education campaigns show marked success to develop national awareness and shift cultural attitudes around reproductive, maternal, newborn and child health (RMNCH), and promote facility-based deliveries. Within the community health worker (CHW) cadre, each village has one RMNCH-focused CHW who conducts PNC visits at the home (*16*). Performance-based financing of healthcare staff has stimulated increased utilization of services as it encourages staff to identify creative ways to conduct services with previously difficult to reach populations due to barriers such as distance (*16*). Rwanda’s use of RapidSMS, an mHealth tool, provides real-time national monitoring to construct relevant, timely programs, and inform cost-effective allocation of the country’s health budget (*20*). In addition, the facility-based maternal death audit helped hospital teams identify causes of death, contributing factors, and make recommendations to reduce reoccurrence (*21*). A cultural shift in the acceptance of facility-based births, improved compensation mechanisms for healthcare staff, data collection mechanisms, and internal audits, joined with the work of skilled birth attendants and CHWs to monitor and connect prospective mothers to antenatal care (ANC) and delivery care, creates an opportunity to improve PNC attendance.

An updated PNC framework was distributed to Rwanda’s health facilities in 2016 and consists of facility-based and home-based PNC visits (**Figure 1**). The package of four visits includes the physical and medical examinations as well as counseling on a variety of topics for the mother and baby.

**Figure 1:**
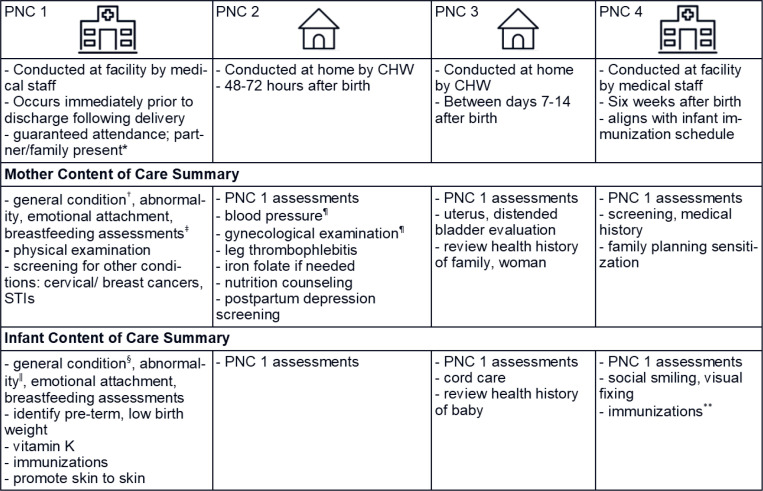
Rwanda’s newly initiated postpartum care framework (*22*–*24*). PNC 2 and 3 are conducted at home because the risk of infection is greater if brought to facility and burden is high for new moms. Rwanda’s model mirrors the 2013 WHO 4 PNC visit guidelines (*9*). *From ref. (*25*). †Mother general condition assessments: physical examination, hygiene/hand washing counseling, breastfeeding support. ‡Breastfeeding assessments and counseling: milk volume, positioning, attachment, mother’s nutritional intake, concerns (e.g. nipple pain), engorgement, and mastitis, and supportive breastfeeding environment at health center. §Infant general condition assessments: physical examination, weight monitoring, immunization confirmation, social smiling, visual fixing, hearing screen. ‖Infant abnormality assessments: jaundice, thrush, nappy rash, constipation, diarrhea, colic, fever. ¶Verified by health center staff if needed. **Immunizations: BCG, OPV, DTP or DTP-HepB-Hib, Pneumococcal Conjugate, Rotavirus (*26*).

Despite the infrastructure support and established importance of PNC, utilization beyond PNC 1 remains low in Rwanda. According to the Rwanda DHS 2014-15 (18 months prior to the study data collection), 43% of mothers and 19% of infants receive a postnatal checkup within two days of delivery as indicated by the guidelines (*19*). In 2016, the Health Management Information System (HMIS) reported national coverage of 60% for PNC 1 (*27*). In spite of the differences in the two data sources, these numbers reflect an increase that is likely to continue. With most of maternal and infant deaths occurring during the first month after birth, guidelines recommend visits beyond this higher risk window with PNC 2, 3 and 4, up to 6 weeks after birth. In our study districts in 2017, the number of PNC 4 visits at 6 weeks was 56% of the number of PNC 1 visits, whereas in 2018 it was 73% of the number of PNC visits (*28*). With a national PNC 1 rate of 60%, this translates to a PNC rate of 34% in 2017 and 44% in 2018 in our study districts. These sources indicate that while PNC attendance is on the rise, it has still not reached optimal levels.

Despite its remarkable achievements in health, barriers still remain for timely uptake of PNC services in Rwanda. As part of a larger study on ANC and PNC in Rwanda, we conducted a qualitative study to better understand the perspectives of providers, administrators, and service users. The objective of this paper is to describe perceptions and attitudes towards PNC services and identify barriers and facilitators of PNC utilization in Rwanda.

## METHODS

This qualitative data was collected as a part the East Africa Preterm Birth Initiative (PTBi-EA) to guide development of a group ANC and PNC model in Rwanda. Key-informant in-depth interviews (IDIs) and focus group discussions (FGDs) were planned for five districts (Bugesera, Burera, Nyarugenge, Rubavu and Nyamasheke) in August 2016. Districts were selected in collaboration with the Ministry of Health (MOH) based on indicators of preterm birth rates and to minimize overlap with other partners. Participants were purposively selected to represent a variety of stakeholders and included district authorities, healthcare service providers, CHWs, and ANC/PNC potential clients. Eligibility criteria required that healthcare providers deliver or supervise ANC or PNC services. Participants were informed of the study objectives and written consent translated in the local language, Kinyarwanda, was obtained from each participant. IDI and FGDs were conducted in person and audio recorded in non-public settings, often in private rooms at health facilities in the local language, Kinyarwanda. Researchers recorded field notes about the discussion content, tone, and context of IDI and FGD activities. Twenty FGDs and 35 IDIs were planned; of these, 16 FGDs with 180 participants and 22 IDIs were completed at the time of thematic saturation, thus data collection was withdrawn for one of the five planned districts (**Table 1**).

**Table 1:** Distribution of respondents from Rwanda’s four participating districts; participants were purposively selected to represent a variety of stakeholders and included district authorities, healthcare service providers, CHWs, and ANC/PNC potential clients

PARTICIPANT LEVEL	NUMBER OF FGDS/IDIS CONDUCTED	NUMBER OF PARTICIPANTS PER DISTRICT/FGD/IDINYARUGENGE BUGESERA BURERA RUBAVU				TOTAL PARTICIPANTS
**Community:**						
CHW	4 FGDs	11	12	12	12	**47**
Women 18-21 years	4 FGDs	7	12	11	10	**40**
Women >21 years	4 FGDs	12	12	12	12	**48**
Male partners >21 years	4 FGDs	10	12	12	11	**45**
**Health center**						
ANC/PNC lead	5 IDIs	1	2	1	1	**5**
Head	4 IDIs	1	1	1	1	**4**
CHW Supervisor	4 IDIs	1	1	1	1	**4**
**District:**						
V/Mayor Social Affairs	1 IDI	-	1	-	-	**1**
Health Director	4 IDIs	1	1	1	1	**4**
CHW supervisor, hospital	4 IDIs	1	1	1	1	**4**
Total	16 FGDs; 22 IDIs					202

FGDs –focus group discussion; IDIs – in-depth interviews; CHW – community health worker; ANC – antenatal care; PNC – postnatal care

Standardized data collection was facilitated by a three-day training for four moderators and four note takers on best practices of FGD and IDIs prior to study initiation; debriefings to close data collection days further ensured team consistency. Study clearance was granted by the Rwanda National Ethics Committee (#633/RNEC/2016) and UCSF Institutional Review Board (16-21177). A team of eight interviewers grouped in pairs facilitated IDIs and FGD across the four districts.

### Semi-structured IDIs

Semi-structured IDIs were conducted with healthcare providers and professionals to determine perceptions of benefits and limitations of current PNC service delivery. At the district-level we interviewed one Vice-Mayor in charge of Social Affairs, four District Directors in charge of Health; and four CHW hospital supervisors. At the health center level, we interviewed five nurses/midwives in charge of ANC and/or PNC, four Heads of Health Centers and four CHW Supervisors. Questions sought to illuminate information about services provided in both the health center and community, explore experiences in PNC service provision, determine how decisions about PNC-seeking behavior influenced attendance, and reasons for not accessing PNC services. IDIs lasted 40 to 60 minutes.

### Focus group discussions

Four types of FGDs were carried out in each of four districts: 1) women between 18-21 years of age; 2) women over 21 years of age; 3) male partners 21 years of age or older of women of reproductive age; and 4) CHW in charge of maternal and child health or *agent de santé maternelle*. CHWs were selected randomly from the health facilities’ catchment area. Monetary incentives to cover round-trip transportation to the discussion location was provided. Two researchers facilitated each FGD of 7-12 participants. During FGDs, participants sat in a semi-circle to facilitate dialogue and were numbered to allow anonymous transcription. FGD topics included ANC and PNC in the same focus group. PNC related questions explored participants’ knowledge, access, visit adherence, barriers to utilization, and areas of improvement. FGDs lasted 90 minutes to two hours.

### Data cleaning and analysis

Audio files were transcribed verbatim. The content analysis approach was used to organize notes and direct quotes were organized into thematic groups using Atlas Ti 7.5.18 (Atkas. ti, Berlin, Germany). Transcripts and field notes were aggregated at the district level and grouped to highlight themes, concepts, convergence, and diverse responses. These results were reviewed by research team members (AM, BRN) to identify key findings related to the study objectives. Representative, verbatim quotes from IDIs and FGDs were selected to illustrate key findings and translated into English for results dissemination. These results were further excerpted to PNC-containing transcripts only; those only addressing topics related to ANC were excluded. These abbreviated transcripts were re-reviewed (PW, NKM) and a code book was developed through two iterations of discussion to ensure comprehensive identification of topics. Once codes and their associate definitions were finalized, two researchers (PW, NKM) applied codes to transcripts in Dedoose 8.0.44. Themes and supporting evidence were created by thorough review of the transcripts, framework, and the relationships captured across transcripts through the applied codes. Codes are listed in **Table 2**.

**Table 2:** Applied codes used to identify themes and supporting evidence by review of transcripts, framework, and relationships across transcripts

1. Lack of sensitization
a. Failure of CHW/provider training model (unaware of service, don’t understand purpose)
b. Failure of health center to educate patients (women are unaware of PNC service, women misunderstand the purpose of PNC service)
2. PNC2+3 available at home, conducted by CHW
3. Incentive to attend PNC (family planning, immunization, mother educated on best practice, birth certificate )
4. Cost barriers: health insurance, distance to health center
5. High workload of healthcare personnel (shortage of staff, provider/patient ratio is too high )
6. Provider attitude towards patient services (respectful care, patient-centered care )
7. Husband and family attitude towards service attendance
8. Patient relationship with CHWs
9. Recommendations to improve attendance

CHW – community health worker; PNC – postnatal care

## RESULTS

Review of interview data revealed four themes highlighting facilitators and barriers to PNC attendance and recommendations to improve participation:

1. There is little awareness of the PNC package in the community; PNC 4 in particular is not well understood;
2. PNC visits by CHWs are well accepted and valued;
3. Providers perceive PNC 4 as an added burden to an already high workload; and
4. Community structures exist to better disseminate key messages about PNC, but have not yet been effectively utilized.

### Theme 1: There is little awareness of the PNC package in the community; PNC 4 in particular is not well understood

FGD and IDI participant responses illustrated a gap in knowledge of PNC. One CHW said, “I do not know that program of returning to the health center” (CHW 11). Patients also expressed they were unaware of the service. One said, “we didn’t know that it was necessary to come back [for PNC]” (Woman 10). Even participants who received PNC services were naive of the label and purpose of the treatment. At “home, the CHW comes and makes a follow up on the baby, they check his/her weight, but they didn’t tell us about PNC” (Woman 6). In this circumstance, even when a patient engages in PNC, the individual is unaware of services within the context of the PNC package.

Despite this lack of knowledge, interviews illustrated that women do return to the health center following delivery, but for the purpose of immunization and family planning services. Despite lack of knowledge about PNC, attendance for these other services following delivery is strong. One CHW recounted, “the woman only comes to get her child vaccinated after one month and a half. The women do not know about the PNC” (CHW 11). The integration of vaccine services into PNC

“should be considered because Rwandan people have not yet understood [PNC]. The only service that they attend is the vaccination of their children…In fact, they do not do a follow up on their health after delivery. We use that opportunity when they have come to seek for vaccinations. We do a follow up on their healthcare.” (CHW Supervisor)

The distribution of postpartum family planning was also cited as an opportunity to engage mothers in PNC. A patient shared that, “even though I had no problem on my side, the nurses or midwives have told me nothing about PNC. Only the CHW told us that we have to come for family planning” (Woman 8). In this circumstance, the patient has been encouraged to return for postpartum family planning, but not for the comprehensive package of services offered during PNC.

### Theme 2: PNC visits by CHWs are well accepted and valued

Participation in PNC includes home visits by the CHW for PNC 2 and 3; numerous testimonies recounted the provision of these services. Following delivery upon arrival at her home, one woman stated that the CHW “came to see me, she check my MUAC [middle upper arm circumference] and she kept following up on my baby until now, inquiring on how he is” (Woman 2). In addition to check-ups for the baby, the mother’s health is also monitored. One patient recounted that she “felt intermittent pain but the CHW treated me and advised me to go back to the health center” (Woman 5). A partner included additional details on the provision of care provided in the home following birth:

“Four days after my wife delivered, the CHW came to visit us, they checked the baby’s weight and found that he increased in weight compared to the birth weight. It was four days after leaving the health center. [The CHW] came back after one week” (Man 10).

Thus, despite an overall report of an absence of PNC attendance at the health center, PNC provision and participation for the home visits is reported by both the CHW and patient populations.

CHWs hold a critical position facilitated by their role as community members and are perceived as trustworthy members of the community. One CHW reflected, “I feel free with her especially when it is the first delivery because she is telling you how she felt the contractions, how she delivered her first baby, how she is happy with it” (CHW 1). A partner also supported these sentiments by explaining, “most of the time, since we are in good relationship with the CHWs, they may come today and come back the following day” (Man 11). Healthcare providers recognize the importance of establishing a relationship by which their client and the community can build trust. One recounted some of her best practices with the provision of care in the community: “before you examine a woman in PNC, you pass through the process of establishing good relationship with your client, and then you register her and do all the measurements written on the file” (Head of Health Center). Another discussed specifically how she establishes a relationship with a new mom:

“You show her a good posture she must have when she breastfeeds her baby and let her know that it is necessary to look at the baby’s face as she breastfeeds. Once you teach all of these things she trusts you and believes that you are well trained to carry out such duties. That moment she cannot hesitate to follow your instructions. She does everything that concerns her healthcare” (CHW 4)

Community members express that CHWs are trusted with the provision of healthcare and that CHWs understand mechanisms by which to successfully establish this relationship to create an environment which enables valued and accepted services.

### Theme 3: Providers perceive PNC 4 as an added burden to an already high workload

The availability, or in some cases, quality, of PNC services may reflect overburdened healthcare personnel. Additional CHWs, nurses, and providers of PNC care were cited as necessary to conduct quality services. One provider stated that additional support is needed for CHWs to include the task of motivating patients to attend: “nurses and doctors from different health centers should help CHWs to motivate people. Everyone in charge of health services and the official who assists the CHWs ought to get involved” (Provider). In this instance, a healthcare center team approach is suggested to encourage PNC attendance. This was further emphasized when a CHW manager stated, “the biggest challenge is that [CHWs] are very busy, [CHWs provide the most] care at the household [level]” (CHW Supervisor). Another provider stated that even maintaining services for ANC was at times not feasible and further impacted the provision of PNC. They stated,

“the program itself is good and it would face no problem if they added other nurses to us. You have seen how it took me long to get the time to talk with you because I was working in more than one service…We are sometimes blamed for delivering bad services while it has been due to the low number of nurses and a lot of work they have.” (Provider)

Additional comments on the impact of insufficient providers on quality of service were observed. A provider shared that “the insufficiency of personnel reflects in all the services” (Provider). A district director also shared personnel shortages as a barrier: “we don’t have sufficient health providers. That may decrease the quality of services offered during ANC or PNC, we cannot perform at a hundred percent” (District Director). The integration of additional services must be considered in conjunction with personnel capacity to ensure successful implementation.

### Theme 4: Community structures exist to better disseminate key messages about PNC, but have not yet been effectively utilized

Participants asserted that increased sensitization during ANC, information dissemination mechanisms, and even the use of disincentives could provide possible methods to improve PNC attendance. FGDs and IDIs provided insights to how health curriculum could be modified to facilitate increased PNC attendance. The means by which women are educated at the health facility can also improve PNC attendance. One woman stated, “I think that the appointment to come back for PNC should be given right during ANC. It is better to teach us about it very early” (Woman 8). Clear communication of expectations during ANC about the postpartum period can facilitate PNC participation. Patient knowledge can be shifted to a continuum of care mentality, and CHWs are well positioned to contribute to this change. One provider said that women “have to know that there are different steps: the pregnancy, the delivery, and PNC. The CHWs ought to urge the women about the other services that they are required to have after delivery at the health center” (Provider).

The use of ANC visits to sensitize women about the importance of PNC 4 at 6 weeks is underutilized. One patient said, “I have always been attending ANC at this health center, and I delivered at this place, but nothing about [PNC] was said until I was discharged” (Patient 7). Thus, utilizing the time that the mother and baby are present at the health center can serve as a prime opportunity to provide PNC. One CHW elaborated that it “all depends on the proper delivery of that knowledge about [PNC]” (CHW 8). Education during ANC could provide needed repeated exposure to encourage PNC attendance. One participant stated that “the most important thing is to look for a motivating factor and to intensify sensitization about use of those services and self-care in general” (CHW Supervisor). A patient’s understanding of the purpose and benefit of PNC services was expressed to be an integral element. One partner supported community

“sensitization before the implementation of this new method. The sensitization aims at encouraging people to understand how useful their involvement will be. They must be aware of its purpose. When people are less informed, they will not [participate] in the new program.” (Man 10)

FGD and IDI participants provided insights into more effective PNC information dissemination mechanisms. Specifically, leveraging men and community leaders, educational material, and media platforms can serve as effective messaging portals. One CHW shared, “when the instructions are given to both the pregnant woman and her husband, the understanding increases and the results thereof. When that is done, after delivery, the husband takes care of his wife” (CHW 8). Inclusion of both parents in PNC information dissemination is critical to encourage use of the service. One CHW stated, “after men have properly understood the usefulness of PNC, they will urge their wives to follow the instructions” (CHW 8), highlighting how the partner’s role could be leveraged for information dissemination. In this statement, the CHW supports the enabling of men as a mechanism to encourage PNC attendance. The involvement of village leaders and families at the time of Rwanda’s monthly mandated community service, Umuganda, was also suggested. Additionally, an introduction “parents’ evening program (akagoroba k’ababyeyi) so that we learn about [PNC]” (Woman 6) was recommended.

Evidence-based public education campaigns, such as a previously successful radio program, was also cited. One participant stated, “I see that [PNC] should be broadcasted and advertised on the radio in the way Urunana drama is presented. Urunana drama is followed by many people” (Woman 7). Previously successful media platforms can be leveraged to increase PNC attendance. As evident from these excerpts, the participants expressed that there are opportunities to improve the way information about PNC can be shared.

Some respondents also suggested disincentives or penalties to motivate PNC attendance. One provider stated that women

“get involved when the program is compulsory. For instance, we know well that the involvement of people in the vaccination for children used to be low. Today, we have a different rate that might be a hundred per cent. What is the reason? The reason is that women fear the charges they are required to pay when they delayed attending.” (Provider)

This provider advocated for the use of previously successful communication strategies to improve patient engagement with services. This provider felt that the imposition of fees for non-compliance had been an effective strategy in the past. One woman stated health authorities must “put much efforts in [encouraging PNC attendance] and sometimes penalties, people will come” (Woman 10). As a patient, this participant states what she perceives as a requirement to motivate the behavior for PNC participation. Attendance for the purpose of retrieving a birth certificate was also suggested as a platform to encourage attendance. One CHW Supervisor elaborated that parents must “come to take [the newborn’s] birth certificate in order to attend, otherwise they stay at home and don’t bring back the babies because they don’t know the importance of PNC” (CHW Supervisor).

## DISCUSSION

A gap in PNC knowledge and acceptance of PNC services exists within the postpartum population. Currently, PNC attendance in Rwanda is neither culturally integrated nor institutionally normalized. New PNC regulations in 2016 were released by the Rwanda MOH, however, the administrative structure to support the execution, as well as encourage the participation of women within this service, is not yet fully realized (*29*).

A shift to attend the offered PNC services is needed to tap into the maternal and child health improvement opportunities. Interviewed participants provided insights for strategies to encourage this behavior change: include significance and expectations of PNC attendance in the ANC curriculum and provide patients with clearly documented expectations for PNC. As expressed by interviewees, the mediums by which government messages are disseminated can influence behavior change at the local level. Local level networks can be leveraged via the empowerment of CHWs and village leaders (Umukuru wu mudugudu) to disseminate information to their respective populations. The utilization of media platforms that have proven successful, such as those used to increase immunization coverage, can engage the population in PNC. Although the use of disincentives was suggested by some interviewees, the use of this motivation medium has led to decreased or late ANC attendance rates and is thus not supported by the authors.

Improvements to the PNC attendance data collection system and service integration can facilitate this shift. Further utilization of the mHealth system in PNC services to track through PNC 4 can serve as a tool to improve adherence and create a true metric for PNC attendance. The inability to accurately measure PNC attendance due to incorrect data collection assumptions is a major barrier. Another strategy to increase participation is to integrate PNC with other clinical services. Insights from the qualitative analysis illuminated possible paths to harness this momentum: 1) staff facilities appropriately to accommodate the provision of PNC, 2) merge 6-week old newborn immunizations with PNC 4 and staff facilities appropriately to accommodate this service, or 3) in addition to merged services, require an approval process on birth certificates that necessitates verification of PNC participation inclusive of immunization completion and postpartum family planning counseling. Bundled services to improve attendance is supported in the literature in the low-middle income country context (*30*).

While the majority of this analysis focused on engagement in PNC after delivery, the entire continuum of care must be considered. Information exposure prior to delivery, such as in ANC, plays an important role in decision-making. Framing around the period of conception, the birth of a child, and a growing family is often clearly demarcated for the ease of reference by the medical profession. However, these phases may not represent the woman’s experience and may not exist as the discrete periods of ANC, birth, and PNC for postpartum women. This concept of the life continuum must be considered in the measurement, evaluation, and improvement in maternal health and the health of the family as a whole (*31*).

## CONCLUSIONS

This qualitative work, conducted close to the time of Rwanda’s new PNC package launch, provides evidence that the PNC package was not initially well understood. The 2016 package revision added additional services, but this analysis shows that the previously existing PNC services were not strongly established at both the health center and population levels. Despite this discordance, we observed that the PNC service delivery performed by CHWs in Rwanda is well-accepted and appreciated by the population, providing assurance that the full package has potential to be well utilized and valued by the population. One potential threat to greater PNC attendance is the perception by providers that the capacity to add additional services is not possible due to a current over-burdened workload. It seems likely this perception could result in attitudes or service delivery organization that discourage attendance for PNC visits at facilities by Rwandan mothers and infants. Additionally, this analysis found that providers and potential users had numerous concrete suggestions to sensitize the population to PNC. These ideas include ways to capitalize on existing community structures and utilize the continuum of care. The new PNC guidelines were implemented over two years prior to this publication and thus it is worthwhile to reevaluate knowledge and attitudes around PNC to illuminate any shifts. Researchers might consider comparing communities with high and low rates of PNC and review the implementation and sensitization strategies used respectively. Further research into rates of PNC coverage required to reduce neonatal mortality would also be of interest.
